# RETRACTION: Is Telephysiotherapy an Option for Improved Quality of Life in Patients With Osteoarthritis of the Knee?

**DOI:** 10.1155/ijta/9879362

**Published:** 2026-08-25

**Authors:** International Journal of Telemedicine and Applications

RETRACTION: A.C. Odole and O.D. Ojo, “Is Telephysiotherapy an Option for Improved Quality of Life in Patients with Osteoarthritis of the Knee?”, *International Journal of Telemedicine and Applications*, vol. 2014 (2014). https://dx.doi.org/10.1155/2014/903816.

The above article, published online on 23 March 2014 in Wiley Online Library (wileyonlinelibrary.com), has been retracted following the agreement of the journal’s Comissioning Editor Nadeem Sarwar; and John Wiley & Sons Ltd.

The retraction has been agreed following an investigation, which identified redundancy with a previously published article by the same author group [1].

The authors were informed of this retraction but did not provide a response.
